# Relationships between different trends of the Mediterranean diet and cardiovascular disease-related risk factors in China: results from the CHNS study, 1997–2009

**DOI:** 10.3389/fnut.2024.1463947

**Published:** 2024-11-08

**Authors:** Zhengyang Bao, Zhengtao Qian, Jiale Chen, Yu Chen, Na Li, Yang Ye, Yongwei Ren, Yiming Tang, Daozhen Chen

**Affiliations:** ^1^Wuxi Maternity and Child Health Care Hospital, Affiliated Women’s Hospital of Jiangnan University, Wuxi, China; ^2^Department of Clinical Laboratory, Changshu Medicine Examination Institute, Changshu, China; ^3^Central Laboratory, Changshu Hospital Affiliated to Soochow University, First People's Hospital of Changshu City, Changshu, China; ^4^Hospital Infection Management Section, Changzhou Wujin Hospital of Traditional Chinese Medicine, Changzhou, China

**Keywords:** cardiovascular diseases, Mediterranean diet, dietary habits, the CHNS study, risk factors

## Abstract

**Objectives:**

The present study aimed to explore the associations between Mediterranean diet trajectories (MDTs) and cardiovascular disease-related risk factors in Chinese adults.

**Methods:**

A total of 4,332 participants from the China Health and Nutrition Survey (CHNS) were included in this study. A group-based trajectory model (GBTM) was used to explore Mediterranean diet trajectories (MDTs). Linear regression analyses were conducted to assess the association between Mediterranean diet trajectories and cardiovascular disease-related risk factors. Stratified analyses by gender were also performed.

**Results:**

The following four groups were identified in the total population: Group 1 (Persistently low MDT, *n* = 292, 6.74%), Group 2 (Descent MDT, *n* = 537, 12.40%), Group 3 (Ascent MDT, *n* = 454, 10.48%), and Group 4 (Persistently high MDT, *n* = 3,049, 70.38%). Compared to the persistently low MDT (Group 1), both Group 2 and Group 4 were negatively related to low-density lipoprotein cholesterol (LDL-C) (Group 2: *β* = −0.23, 95% CI: −0.36 to −0.09, *p* = 0.001; Group 4: *β* = −0.25, 95% CI: −0.37 to −0.14, *p* < 0.001), total cholesterol (TC) (Group 2: *β* = −0.18, 95% CI: −0.32 to −0.04, *p* = 0.010; Group 4: *β* = −0.31, 95% CI: −0.43 to −0.19, *p* < 0.001), and uric acid (UA) (Group 2: *β* = −15.16, 95% CI: −28.66 to −2.56, *p* = 0.019; Group 4: *β* = −27.51, 95% CI: −38.77 to −16.25, *p* < 0.001) after adjusting for potential risk factors. Only Group 4 exhibited a negative relationship with triglycerides (TG) (*β* = −0.18, 95% CI: −0.34 to −0.02, *p* = 0.028) and blood glucose (*β* = −0.19, 95% CI: −0.37 to −0.02, *p* = 0.032).

**Conclusion:**

Four MDTs were identified among the total participants, including men and women. These trajectories were summarized as persistently low MDT, ascent MDT, descent MDT, and persistently high MDT. Adherence to high MDTs could reduce the level of some CVD-related risk factors. The impact of the remote MD on CVD-related risk factors may be greater than that of the proximal MD.

## 1 Introduction

Cardiovascular diseases (CVDs) are a global health concern and the leading cause of death in China (1), accounting for approximately 40% of deaths in the Chinese population (2). An increasing number of adults survive into old age with chronic CVDs after experiencing cardiovascular events that would have previously resulted in death at a younger age. Simultaneously, comorbid CVDs and other conditions may accelerate the decline in quality of life in later years (3).

Hyperlipidemia usually refers to an increase in triglycerides (TG) and/or total cholesterol (TC) in the plasma, as well as an increase in low-density lipoprotein cholesterol (LDL-C) and a decrease in high-density lipoprotein cholesterol (HDL-C). Imbalanced blood levels of TG, TC, LDL-C, or HDL-C could increase the risk of myocardial infarction, stroke, atherosclerotic cardiovascular disease (ASCVD), and other conditions (4). It has been reported that people with hyperlipidemia are roughly twice as likely to develop CVDs compared to those with normal TG levels (5).

Diabetes is a group of metabolic disorders caused by absolute or relative insufficiency of insulin secretion or issues with insulin utilization, with hyperglycemia as the primary indicator. Both developed and developing countries experience a dramatic increase in the prevalence of diabetes. Adults with diabetes have a 2- to 4-fold increased risk of cardiovascular diseases compared to those without diabetes, and this risk rises with worsening glycemic control (6).

Hyperuricemia is a chronic metabolic condition caused by purine metabolism disorders. In the circulatory system, purine metabolism yields uric acid as its final oxidation product. Some studies have indicated that serum acid levels are independently and significantly associated with the risk of cardiovascular mortality (7, 8). Uric acid (UA) levels are often useful as an indicative marker for predicting CVDs.

The Mediterranean diet (MD) is characterized by a high intake of olive oil, nuts, cereals, fruits, and vegetables; a moderate intake of fish, poultry, and wine with meals; and a low intake of red and processed meats, dairy products, and sweets (9). The MD serves as a healthy dietary pattern to prevent major cardiovascular events in adults (10). Although quite a few studies have demonstrated that the MD plays an important role in protecting against the development of hyperlipidemia (11), diabetes (12), and hyperuricemia (13), a relevant study showed a limited effect of diet patterns on controlling serum uric acid (SUA) levels (14). The insufficient evidence regarding the impact of long-term MD habits on cardiovascular-related serological indicators in adulthood, such as TG, TC, LDL-C, HDL-C, glucose, and UA, still requires further research.

Therefore, the present study aimed to (1) explore the long-term trends of the MD in the Chinese population, including differences by gender, from 1997 to 2009; (2) assess the association between MD trends and CVD-related risk factors; and (3) determine the significance of the effects of the remote versus proximal MD on CVD-related risk factors.

## 2 Materials and methods

### 2.1 Study design and population

The China Health and Nutrition Survey (CHNS) used a multistage, random cluster sampling process to obtain samples from 15 Chinese provinces, aiming to examine the effects of health, nutrition, and family planning policies or programs. More detailed information about the design and methods of the CHNS can be found elsewhere (15). Fasting blood samples were first collected in 2009. All biological sample collection, processing, preservation, and related analytical tests were conducted by trained professionals, who effectively ensured quality control throughout the sample collection process.

**Figure 1:**
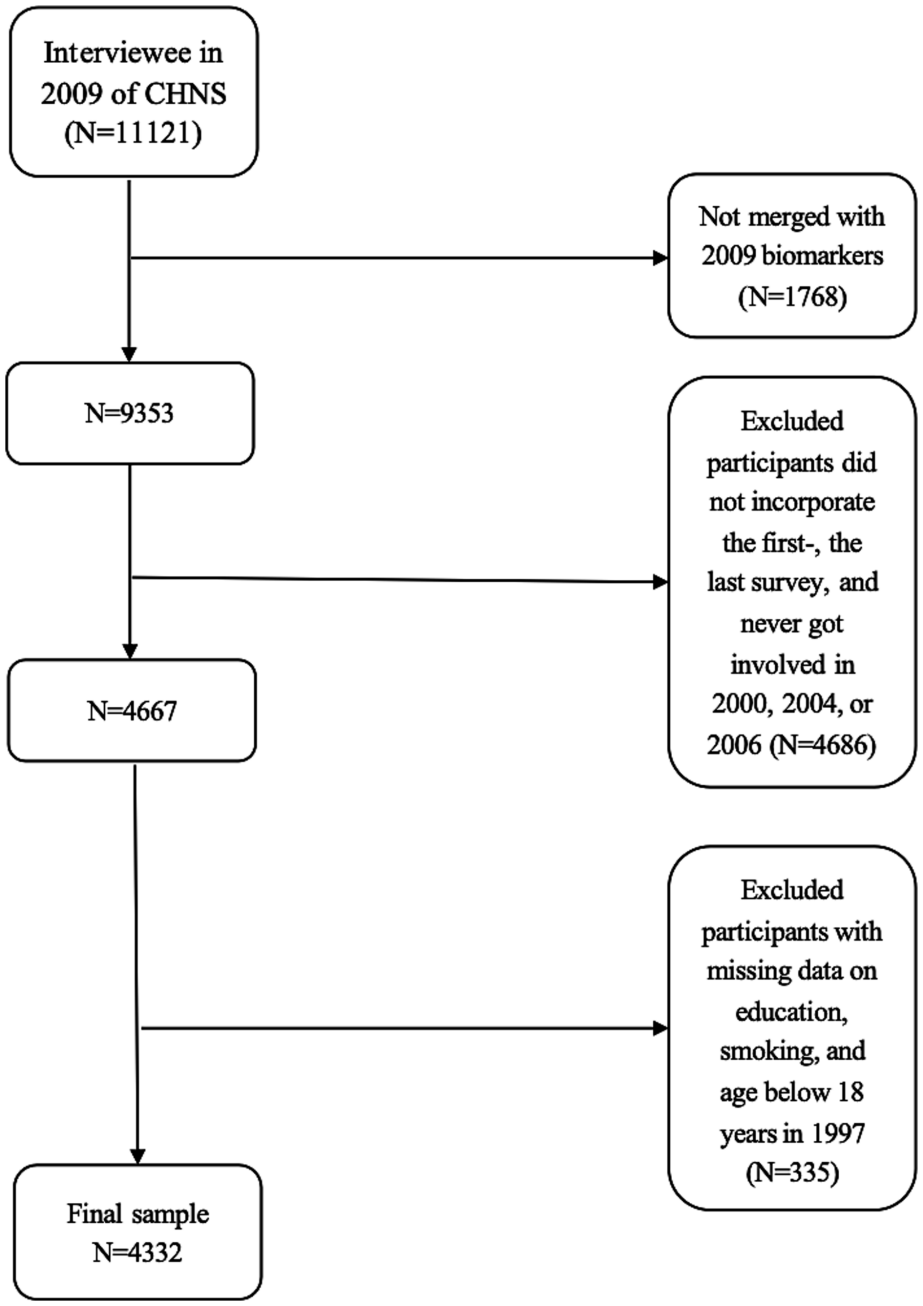
Flow chart of the analytic sample.

As data on biological samples were only available in 2009, the present study population was included based on the 2009 survey data. A total of 11,121 participants were interviewed in the 2009 CHNS survey, of whom 1,768 did not undergo fasting blood tests. The evaluation of the trajectory of the Mediterranean diet requires at least three times the survey data. Therefore, this study used data from the CHNS conducted between 1997 and 2009, ensuring that participants included responses from the first survey, the last survey, and at least one survey from 2000, 2004, or 2006 (*N* = 4,667). Participants with incomplete information on education and smoking status as well as those under 18 years of age in 1997 were excluded (*N* = 335). Finally, 4,332 participants (2,044 men and 2,288 women) remained in the final data analysis. The sample selection flowchart of the current analysis is presented in Figure 1.

### 2.2 Assessment of the Mediterranean diet

The Mediterranean diet (MD) was assessed based on daily dietary intake using 3-day and 24-h dietary recall. The type and amount of food consumed per meal were recorded using the direct weighing method during the survey. More detailed information on dietary intake collection can found be in previous reports (16). The MD score scale proposed by Trichopoulou was used in the present study. A total of nine components were included in the scale, comprising vegetables, legumes, fruits, nuts, cereals, fish, meat and meat products, dairy products, and alcohol. Gender-specific median values for seven components (excluding dairy products and alcohol) were used as cutoffs. The individuals whose intake was at or above the cutoff were assigned a value of 1, while those whose intake was below the cutoff were assigned a value of 0 for six components—vegetables, legumes, fruits, nuts, cereals, and fish—that were presumed to be beneficial. The method of assigning values to meat was opposite to the approach used for the other components. For alcohol intake, a value of 1 was assigned to the women who consumed 5–25 g/day and the men who consumed 10–50 g/day, while a value of 0 was assigned to all others. For the dairy category, a value of 1 was assigned to the participants whose dairy product intake was 5–25 g/day, while a value of 0 was assigned to others. The MD score was calculated by summing the scores from the nine categories, which ranged from 0 to 9.

### 2.3 Assessment of cardiovascular disease-related risk factors

A blood sample of 12 mL (in three 4 mL tubes) was collected after overnight fasting. Before sending the samples to the laboratory, inspectors were responsible for verifying the basic information of the volunteers to facilitate further investigation. Blood samples need to be stored in a refrigerator, with a ratio of sample to dry ice of 1:1, and transferred to the local laboratory for testing within 3 h. High-density lipoprotein (HDL, mmol/L) cholesterol and low-density lipoprotein (LDL, mmol/L) cholesterol were measured using an enzymatic method (Hitachi 7,600, Kyowa, Japan). Triglyceride (TG mmol/L) and total cholesterol (TC mmol/L) were measured using GPO-PAP (Hitachi 7,600, Kyowa, Japan) and CHOD-PAP (Hitachi 7,600, Kyowa, Japan) methods, respectively. Glucose (mmol/L) was measured using the GOD-PAP (Hitachi 7,600, Randox, UK) method. Hemoglobin A1c (HbA1c, %) was measured using HLC (HLC-723 G7, Tosoh, Japan, or D10, Bio-Rad, USA) or HPLC (PDQ A1c, Primus, USA) with whole fresh blood. Serum uric acid (SUA, mg/dL) levels were measured using an enzymatic colorimetric method (Hitachi 7,600, Randox, UK). All of these blood indexes were considered continuous variables.

### 2.4 Assessment of other covariates

Sociodemographic and health-related variables were obtained from the general questionnaire. The sociodemographic factors included age (continuous), gender (men and women), marital status (spinsterhood, married, divorced, and others), educational level (none/primary school, middle school, and high school+), and place of residence (urban or rural area). The health-related information included physical activity, smoking status (never, former smoker, and current smoker), history of heart attack (yes/no), and body mass index (BMI). Physical activity was evaluated across four domains: occupational activities, transportation activities, recreational activities, and sedentary activities. Physical activity was measured by the metabolic equivalent of task (MET) hours per week. The BMI was calculated as weight in kilograms divided by height in meters squared.

### 2.5 Statistical analysis

All statistical analyses were conducted using STATA version 16.0 (Stata Corp, College Station, Texas). A group-based trajectory model (GBTM) was used to identify groups of individuals following similar developmental Mediterranean diet trajectories (MDTs). The survey years from 1997 to 2009 were used as a timescale for the GBTM. The “Traj” package in STATA was used to conduct the Mediterranean diet trajectory (MDT) analysis with a cnorm type among the participants in the five surveys. A two-stage approach was employed to complete the establishment process of the MDT analysis. First, model fitting was conducted in sequence by grouping trajectories from number one to number five based on a cubic form for each trajectory. The lowest absolute value of the Bayesian information criterion (BIC) was determined as the most optimal number of the MDTs. Second, the BIC values and the plot of the best-fitting number of the MDTs were combined to determine the most appropriate polynomial order for each trajectory, which could be linear, quadratic, or cubic. Some evaluation principles included the following: average posterior probability of assignment (APPA) values >70%, odds of correct classification (OCC) > 5.0 (17), and estimated group proportions (EGPs) that were close to the corresponding assigned group proportions (AGPs). Thus, each participant was assigned to the corresponding Mediterranean diet trajectory group based on maximum likelihood estimation, which allowed us to analyze the association between the MDTs and cardiovascular disease-related risk factors. Linear regression analyses were performed to assess this relationship. Gender-stratified analyses were also performed to assess the effect of gender. The data were presented as means ± standard deviations (SDs) for continuous variables or as frequencies with percentages for categorical variables in the 2009 survey for descriptive analysis. Pearson’s *x*^2^ test was performed to compare the distribution of categorical variables across different Mediterranean diet groups. Differences among the four groups were compared using one-way ANOVA for continuous variables. A two-tailed *p* < 0.05 was considered significant.

## 3 Results

### 3.1 Trajectories of the Mediterranean diet

**Figure 2:**
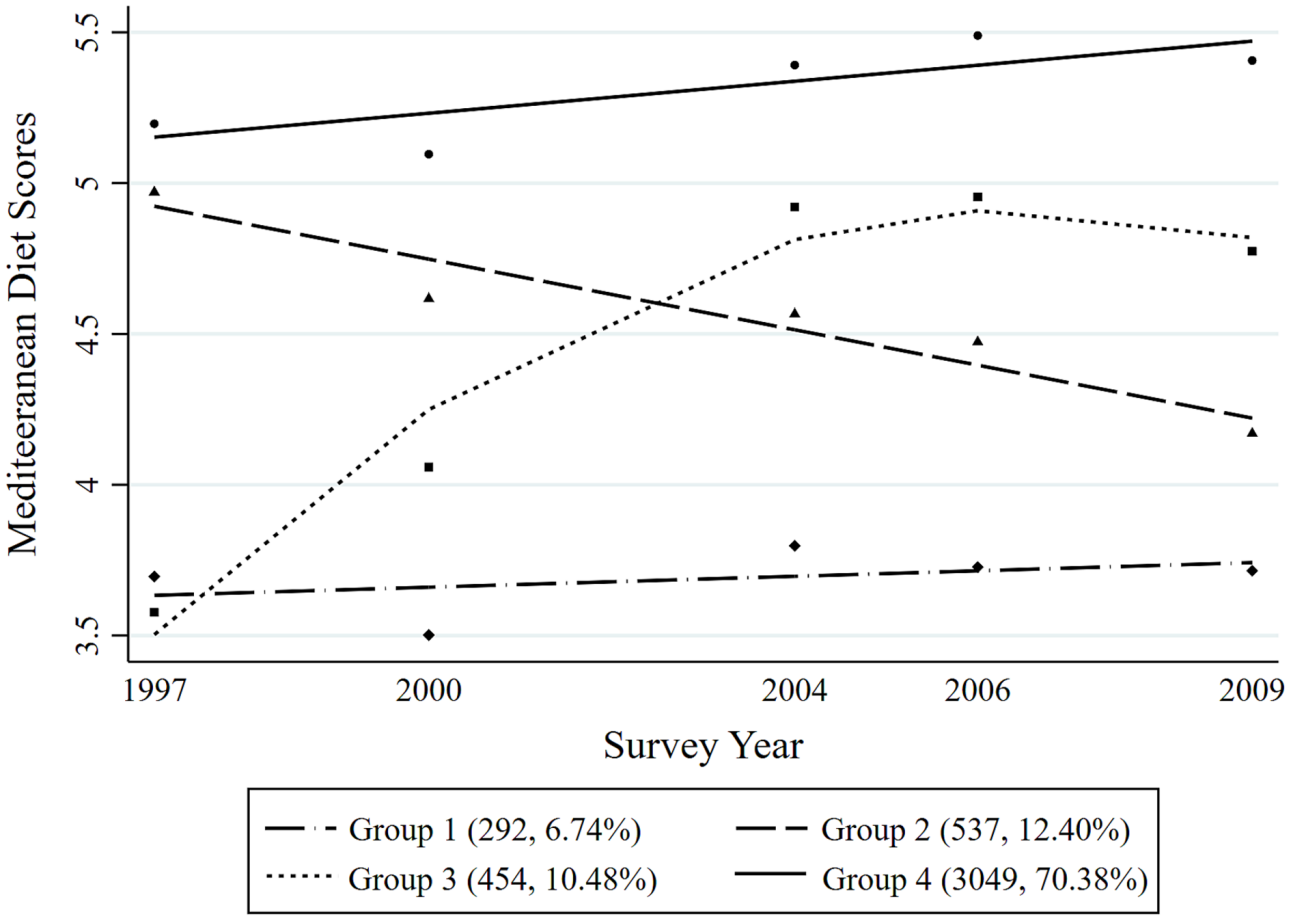
Mediterranean diet trajectories from 1997 to 2009 among the Chinese adults.

**Figure 3:**
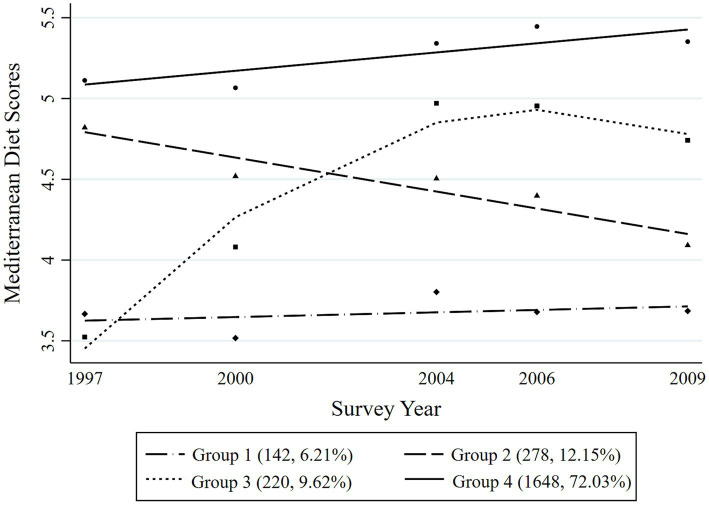
Mediterranean diet trajectories from 1997 to 2009 among the adult men in the Chinese population.

**Figure 4:**
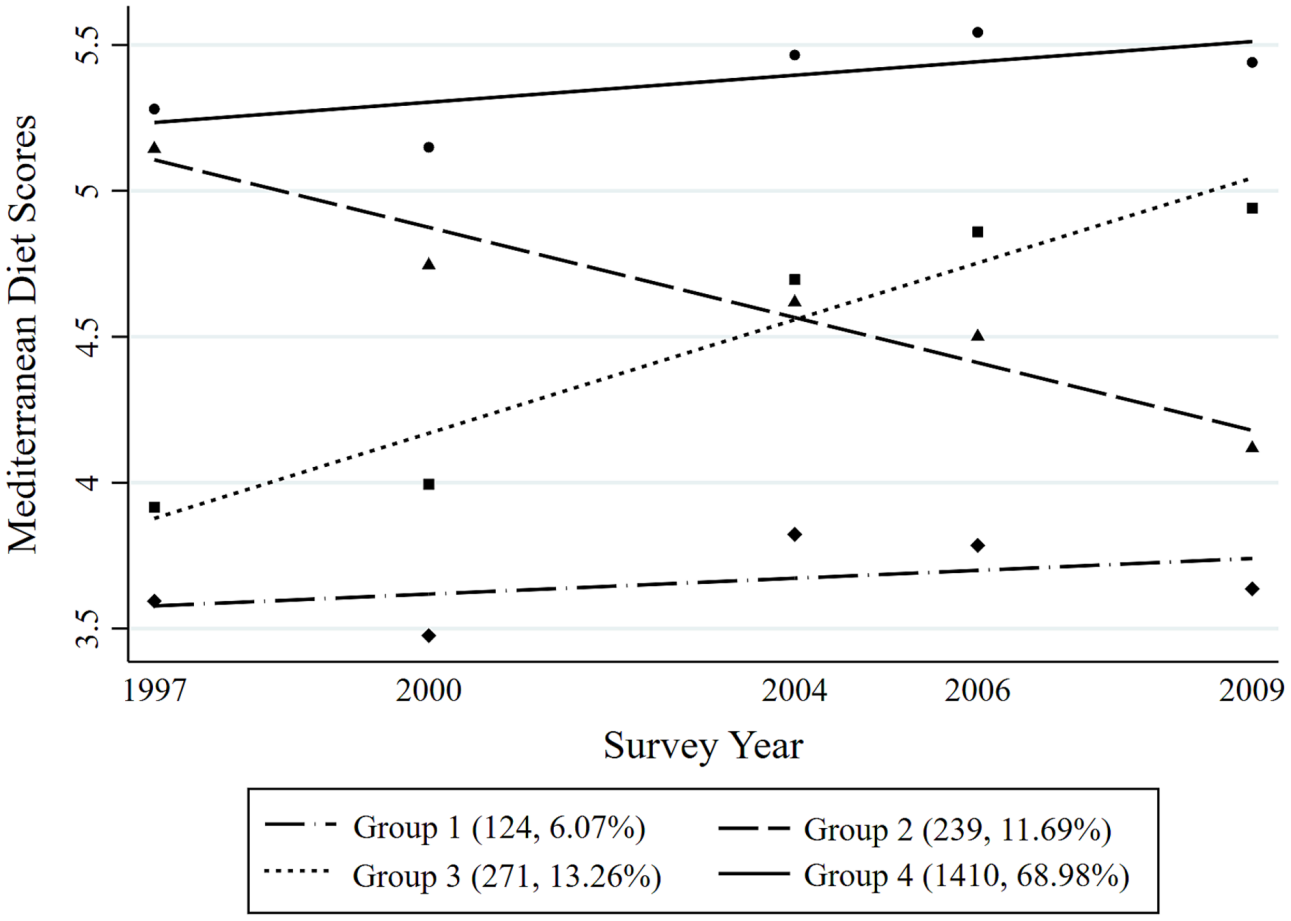
Mediterranean diet trajectories from 1997 to 2009 among the adult women in the Chinese population.

Four distinct MDTs were identified during the 12-year period among the overall participants (Figure 2). Group 1 (Persistently low MDT, *n* = 292, 6.74%) included the participants with the consistently lowest Mediterranean diet scores during the survey years; Group 2 (Descent MDT, *n* = 537, 12.40%) included those who initially had higher Mediterranean diet scores but then experienced a significant decrease robustly; Group 3 (Ascent MDT, *n* = 454, 10.48%) included those who started with low Mediterranean diet scores at the first survey point and then experienced a rapid increase in their scores over a time until they reached a stable level; and Group 4 (Persistently high MDT, *n* = 3,049, 70.38%) included those who consistently had the highest Mediterranean diet scores with a slow upward trend in the following years. The four different MDTs were distinguished among both men and women during the survey period, as presented in Figures 3, 4, respectively. In men, Group 1 (Persistently low MDT, *n* = 142, 6.21%), Group 2 (Descent MDT, initially high and rapidly descended, *n* = 278, 12.15%), Group 3 (Ascent MDT, initially low with a sharp upward trend that then stabilized, *n* = 220, 9.62%), and Group 4 (Persistently high MDT, *n* = 1,648, 72.03%) were found. In the women, Group 1 (Persistently low MDT, *n* = 124, 6.07%), Group 2 (Descent MDT, initially high and rapidly descended, *n* = 239, 11.69%), Group 3 (Ascent MDT, initially low with a sharp upward trend, *n* = 271, 13.26%), and Group 4 (Persistently high MDT, *n* = 1,410, 68.98%) were found. The process for estimating the MD trajectories of the participants is shown in Supplementary Tables S1, S2.

### 3.2 Characteristics of participants according to the MDTs

The general characteristics of the participants from the 2009 survey across the four trajectories of the Mediterranean diet are shown in Table 1. The participants in the persistently high MDT group tended to be younger (54.96 ± 11.78 years), men, non-smokers, with a primary education level, married, having a higher BMI, engaging in more MET, having a higher probability of developing a heart attack, residing in rural regions, and having the lowest levels of LDL, TG, TC, glucose, HbA1c, and UA.

**Table 1:** Basic characteristics.

Group	Group 1 (Persistently low MDT)	Group 2 (Descent MDT)	Group 3 (Ascent MDT)	Group 4 (Persistently high MDT)	*P*-value
*N*	292	537	454	3,049	
Age	56.20 ± 13.89	55.80 ± 13.60	56.79 ± 12.98	54.96 ± 11.78	0.010*
Gender %					0.244
Men	130 (44.52%)	243 (45.25%)	202 (44.49%)	1,469 (48.18%)	
Women	162 (55.48%)	294 (54.75%)	252 (55.51%)	1,580 (51.82%)	
Smoking status %					0.064
Never smoking	215 (73.63%)	365 (67.97%)	319 (70.26%)	2,044 (67.04%)	
Ever smoking	8 (2.74%)	27 (5.03%)	15 (3.30%)	99 (3.25%)	
Still smoking	69 (23.63%)	145 (27%)	120 (26.43%)	906 (29.71%)	
Education %					<0.001*
None/Primary	115 (39.38%)	261 (48.6%)	227 (50.00%)	1,765 (57.89%)	
Moderate	162 (55.48%)	254 (47.3%)	209 (46.04%)	1,234 (40.47%)	
High+	15 (5.14%)	22 (4.10%)	18 (3.96%)	50 (1.64%)	
Marital status %					0.124
Spinsterhood	3 (1.03%)	5 (0.93%)	3 (0.66%)	29 (0.95%)	
Married	247 (84.59%)	452 (84.17%)	400 (88.11%)	2,689 (88.19%)	
Divorced and others	42 (14.38%)	80 (14.9%)	51 (11.23%)	331 (10.86%)	
BMI	23.21 ± 3.16	23.39 ± 3.49	23.38 ± 3.24	23.39 ± 3.49	0.86
MET	78.06 ± 81.98	84.86 ± 88.92	85.96 ± 116.01	106.00 ± 100.23	<0.001*
Heart attack %	1 (0.34%)	9 (1.68%)	4 (0.88%)	29 (0.95%)	0.271
Region %					<0.001*
Urban	171 (58.56%)	230 (42.83%)	177 (38.99%)	671 (22.01%)	
Rural	121 (41.44%)	307 (57.17%)	277 (61.01%)	2,378 (77.99%)	
HDL-C mmol/L	1.47 ± 0.61	1.47 ± 0.69	1.43 ± 0.51	1.46 ± 0.44	0.527
LDL-C mmol/L	3.22 ± 1.05	3.00 ± 0.96	3.13 ± 0.98	2.98 ± 0.94	<0.001*
TG mmol/L	1.73 ± 1.29	1.77 ± 1.67	1.82 ± 1.59	1.60 ± 1.26	<0.001*
TC mmol/L	5.14 ± 1.15	4.97 ± 1.02	5.07 ± 1.03	4.87 ± 0.96	<0.001*

MDT, the Mediterranean diet trajectory; BMI, body mass index; MET. Metabolic equivalent of task; HDL-C, high density lipoprotein; LDL-C, low density lipoprotein; TG, triglyceride; and TC, total cholesterol. *p < 0.05.

### 3.3 The association between the MDTs and cardiovascular disease-related risk factor*s*

The association between the MDTs and cardiovascular disease-related risk factors among the overall population is presented in Table 2. Compared to the Group 1 (persistently low MDT), both Group 2 and Group 4 were negatively related to LDL (Group 2: *β* = −0.23, 95% CI: −0.36 to −0.09, *p* = 0.001; Group 4: *β* = −0.25, 95% CI: −0.37 to −0.14, *p* < 0.001), TC (Group 2: *β* = −0.18, 95% CI: −0.32 to −0.04, *p* = 0.010; Group 4: *β* = −0.31, 95% CI: −0.43 to −0.19, *p* < 0.001), and UA (Group 2: *β* = −15.16, 95% CI: 0.-28.66 to −2.56, *p* = 0.019; Group 4: *β* = −27.51, 95% CI: 0.-38.77 to −16.25, *p* < 0.001) after adjusting for the potential risk factors. Only Group 4 had a negative relationship with TG (*β* = −0.18, 95% CI: −0.34 to −0.02, *p* = 0.028) and glucose (*β* = −0.19, 95% CI: −0.37 to −0.02, *p* = 0.032). Among men, only the persistently high MDT (Group 4) was negatively associated with LDL (*β* = −0.32, 95% CI: −0.50 to −0.14, *p* < 0.001), TG (*β* = −0.35, 95% CI: −0.63 to −0.08, *p* = 0.011), TC (*β* = −0.46, 95% CI: −0.64 to −0.29, *p* < 0.001), and UA (*β* = −29.75, 95% CI: 0.-48.99 to −10.51, *p* = 0.002) using the persistently low MDT as the reference group (Table 3). Among women, Group 2 and Group 4 were negatively related to LDL (Group 2: *β* = −0.29, 95% CI: −0.48 to −0.10, *p* = 0.003; Group 4: *β* = −0.24, 95% CI: −0.41 to −0.08, *p* = 0.004) and UA (Group 2: *β* = −21.32, 95% CI: 0.-37.17 to −5.46, *p* = 0.010; Group 4: *β* = −23.98, 95% CI: 0.-37.74 to −10.22, *p* = 0.001) when using the persistently low MDT (Group 1) as the reference group. Only Group 4 had a negative relationship with TC (*β* = −0.21, 95% CI: −0.39 to −0.04, *p* = 0.015) compared to Group 1 among women (Table 4).

**Table 2:** The association of the Mediterranean diet trajectories and cardiovascular disease-related risk factors.

Outcomes	Group 1	Group 2		Group 3		Group 4	
		*β* (95% CI)	*P*-value	*β* (95% CI)	*P*-value	*β* (95% CI)	*P*-value
HDL-C	ref.	0.01 (−0.06, 0.08)	0.743	−0.04 (−0.11, 0.03)	0.271	−0.01 (−0.07, 0.05)	0.635
LDL-C	ref.	**−0.23 (−0.36, −0. 09)**	0.001*	−0.12 (−0.26, 0.02)	0.084	**−0.25 (−0.37, −0. 14)**	<0.001*
TG	ref.	0.003 (−0.18, 0.19)	0.977	0.06 (−0.14, 0.25)	0.574	**−0.18 (−0.34, −0. 02)**	0.028*
TC	ref.	**−0.18 (−0.32, −0. 04)**	0.010*	−0.11 (−0.25, 0.03)	0.135	**−0.31 (−0.43, −0.19)**	<0.001*
Blood Glucose	ref.	0.05 (−0.15, 0.25)	0.626	0.06 (−0.14, 0.27)	0.547	**−0. 19 (−0.37, −0.02)**	0.032*
HbA1c	ref.	−0.02 (−0.15, 0.12)	0.801	−0. 02 (−0.16, 0.11)	0.728	−0.07 (−0.18, 0.05)	0.254
UA	ref.	**−15.61 (−28.66, -2.56)**	0.019*	−7.24 (−20.7, 6.21)	0.291	**−27.51 (−38.77, -16.25)**	<0.001*

*β*, coefficient; CI, confidence interval; HDL-C, high density lipoprotein; LDL-C, low density lipoprotein; TG, triglyceride; and TC, total cholesterol. Controlling for potential risk factors: age, gender, smoking status, education level, marital status, BMI, MET, history of heart attack, and region. The bold values indicated a statistically significant relationship between the Mediterranean diet trajectory and outcomes. *p < 0.05.

**Table 3:** The association between Mediterranean diet trajectories and cardiovascular disease-related risk factors in men.

Outcomes	Group 1	Group 2		Group 3		Group 4	
		*β* (95% CI)	*P*-value	*β* (95% CI)	*P*-value	*β* (95% CI)	*P-*value
HDL-C	Ref.	−0.04 (−0.15, 0.06)	0.433	−0.04 (−0.14, 0.07)	0.482	−0.0002 (−0.896, 0.891)	0.996
LDL-C	Ref.	−0.17 (−0.37, 0.04)	0.114	−0.15 (−0.35, 0.05)	0.144	**−0.32 (−0.50, −0. 14)**	<0.001*
TG	Ref.	−0.04 (−0.36, 0.27)	0.786	0.01 (−0.30, 0.33)	0.934	**−0.35 (−0.63, −0. 08)**	0.011*
TC	Ref.	−0.20 (−0.40, 0.01)	0.057	−0.20 (−0.39, 0.003)	0.054	**−0.46 (−0.64, −0. 29)**	<0.001*
Blood Glucose	Ref.	0.24 (−0.09, 0.57)	0.153	0.15 (−0.18, 0.47)	0.371	−0.12 (−0.40, 0.17)	0.422
HbA1c	Ref.	0.05 (−0.14, 0.24)	0.582	−0.01 (−0.19, 0.18)	0.937	−0.03 (−0.20, 0.13)	0.691
UA	Ref.	−10.95 (−33.27, 11.37)	0.336	3.07 (−18.79, 24.94)	0.783	**−29.75 (−48.99, -10.51)**	0.002*

*β*, coefficient; CI, confidence interval; HDL-C, high density lipoprotein; LDL-C, low density lipoprotein; TG, triglyceride; and TC, total cholesterol. Controlling for potential risk factors: age, smoking status, education level, marital status, BMI, MET, history of heart attack, and region. The bold values indicated a statistically significant relationship between the Mediterranean diet trajectory and outcomes. *p < 0.05.

**Table 4:** The association between Mediterranean diet trajectories and cardiovascular disease-related risk factors in women.

Outcomes	Group 1	Group 2		Group 3		Group 4	
		*β* (95% CI)	*P*-value	*β* (95% CI)	*P*-value	*β* (95% CI)	*P*-value
HDL-C	Ref.	0.02 (−0.08, 0.13)	0.645	−0.09 (−0.19, 0.02)	0.104	−0.06 (−0.15, 0.03)	0.173
LDL-C	Ref.	**−0.29 (−0.48, −0. 10)**	0.003*	−0.19 (−0.38, 0.01)	0.064	**−0.24 (−0.41, −0. 08)**	0.004*
TG	Ref.	0.04 (−0.19, 0.27)	0.733	0.12 (−0.12, 0.35)	0.333	0.03 (−0.17, 0.23)	0.774
TC	Ref.	−0.20 (−0.4, 0.003)	0.053	−0.13 (−0.34, 0.08)	0.221	**−0.21 (−0.39, −0. 04)**	0.015*
Blood Glucose	Ref.	−0.06 (−0.33, 0.21)	0.668	0.02 (−0.26, 0.3)	0.89	−0.22 (−0.45, 0.01)	0.06
HbA1c	Ref.	−0.02 (−0.22, 0.18)	0.842	−0.01 (−0.22, 0.2)	0.922	−0.07 (−0.24, 0.1)	0.435
UA	Ref.	**−21.32 (−37.17, -5.46)**	0.008*	−12.83 (−29.36, 3.7)	0.128	**−23.98 (−37.74, -10.22)**	0.001*

*β*, coefficient; CI, confidence interval; HDL-C, high density lipoprotein; LDL-C, low density lipoprotein; TG, triglyceride; and TC, total cholesterol. Controlling for potential risk factors: age, smoking status, education level, marital status, BMI, MET, history of heart attack, and region. The bold values indicated a statistically significant relationship between the Mediterranean diet trajectory and outcomes. *p < 0.05.

## 4 Discussion

To the best of our knowledge, this is the first study to report the association between Mediterranean diet patterns and CVD-related risk factors in China using a large cohort followed for 12 years. This study has documented three major findings. First, four MDTs were found among all participants, both men and women, summarized as persistently low MDT, ascent MDT, descent MDT, and persistently high MDT. However, the trend of the ascent MDT in the women was more gradual than that in the men. Second, in both the overall group of participants and among men specifically, the persistently high MDT was associated with reduced levels of LDL-C, TG, TC, glucose, and UA. The descent MDT led to reduced LDL-C, TC, and UA levels in the total participants. In women, the persistently high and descent MDTs were associated with decreases in LDL-C and UA levels. Only the persistently high MDT was associated with reduced TC levels in the women. Third, in the above three groups, the ascent MDT did not affect CVD-related risk factors among all the participants. In addition, the effect of the remote MD may be greater than that of the proximal MD.

The distributions of the Chinese MDTs were classified into four distinct groups. Except for the ascent MDT, which increased cubically over time in both the total participants and women, and the persistently high MDT, which increased quadratically over time in total population, the other MDT groups increased linearly with time. Typically, MDT scores above six are indicative of a high-quality MD habit (18). The scores of the MDTs in our study were all below six during the survey period, with the population of the persistently high MDT group accounting for a significant proportion. This demonstrates that the diet pattern in China needs further improvement. The trend of the ascent MDT in women was more gradual compared to men. A 12-week nutritional program suggested that men were more susceptible to changes in diet habits than women (19). The proportion of the persistently high MDT in the women was higher than in men. A study involving HIV-infected individuals showed men were more likely to consume meat rather than cereals and vegetables compared to women, concluding that men with HIV have poorer dietary habits than the women (20). The gender difference in long-term diet patterns is not well understood currently. Thus, further studies need to be conducted to explore the persistent diet discrepancies by gender.

Many studies have documented the relationship between the Mediterranean diet and a lower risk of developing CVDs (10, 21, 22). Our study provides evidence that the long-term MDT reduces the risk of cardiovascular disease by lowering CVD-related risk factors such as blood lipid, glucose, and uric acid levels. Greater adherence to the MD correlates with a better lipid profile characterized by lower levels of HDL-C, TC, and TG, which is a finding that is strongly supported by a meta-analysis (23). Moreover, a cross-sectional study involving a sample of 2,044 participants showed that higher adherence to MD was inversely associated with dyslipidemia (24). Several randomized controlled trials also demonstrated the effectiveness of the MD in reducing total and LDL-C levels (25, 26). Nevertheless, an observational study lacked evidence of a significant association with blood lipid abnormalities (27). One reason could be that some studies were conducted in non-Mediterranean countries, meaning that the evaluated MD was not a traditional MD (28).

A significant number of studies have demonstrated a relationship between adherence to the MD and a decrease in the risk of type 2 diabetes (T2D) (29, 30). A meta-analysis including eight cohort studies with a total of 122,810 participants found that higher adherence to the MD could reduce the risk of developing T2D by 19%, highlighting the long-term protective effects of the MD against T2D (29). Another meta-analysis comprising one clinical trial and nine prospective studies displayed that individuals with the highest adherence questionnaire score for the MD may have a 23% lower risk of developing T2D compared to those with the lowest score (30). The results of the aforementioned research strongly support our viewpoint. The association between high adherence to the MD and a lower risk of hyperuricemia is well documented (31, 32). An ATTICA study consisting of 2,380 individuals without cardiovascular or renal diseases found that the MD score was inversely associated with UA levels and could reduce the likelihood of developing hyperuricemia (33).

From a mechanistic point of view, the Mediterranean diet contains many anti-inflammatory ingredients. Omega-3 (*ω*-3) polyunsaturated fatty acids (PUFAs), particularly docosahexaenoic acid (DHA) and eicosapentaenoic acid (EPA), can alter lipid and hemostatic factors, such as platelet aggregation and bleeding time, thereby reducing the risk of CVDs (34). Part of the effect of the MD on CVD prevention may be attributed to polyphenols, which exhibit antioxidant (35), anti-carcinogenic (36), anti-inflammatory (37), hypotensive (38), and even anticoagulant properties (39). In addition, the benefits of dietary fiber in lowering lipid and cholesterol levels have also been reported (40). Dietary fiber includes soluble fiber, which is fermented by bacteria into short-chain fatty acids (SCFAs) in the large intestine. The production of SCFAs leads to alterations in the intestinal microbiota, contributing to the hypocholesterolemic effects of soluble fiber (41). Simultaneously, the MD also includes many other antioxidant components. Antioxidants are compounds that protect cells from the damaging effects of reactive oxygen species (ROS) (42). When ROS-generating reactions are excessively activated, it leads to an imbalance between ROS and antioxidants. Oxidative stress has been linked to cardiovascular disease and diabetes (42). The MD reduces insulin resistance, lowers systolic and diastolic blood pressure, and decreases levels of intercellular adhesion and vascular molecule-1, C-reactive protein, interleukin (IL)-6, and IL-18 (25), as well as UA levels (33), contributing to a decreased risk of CVDs.

The difference in the association between the MDTs and CVD-related risk factors among women and men may be due to gender discrepancies in long-term adherence to the MD. In addition, the cardio-protective role of estrogen may be another reason for this gender difference (43). The ascent MDT was not linked to the CVD-related risk factors in the present study, while the descent MDT showed an inverse relationship. This suggests that the effect of the remote MD on CVD-related risk factors may be greater than the effect of the proximal MD. Therefore, longer adherence to MD habits may have a stronger influence on preventing CVDs.

One limitation of our study is that the CVD-related risk factors were only collected in 2009. Therefore, the possibility of the reverse effect of the CVD-related risk factors on the diet cannot be excluded. Another limitation is that a certain degree of recall bias might have had an influence on the research results because the analyzed data were collected through questionnaires. Meanwhile, the dietary structure of the Chinese population is different from that of other regions, which might have influenced the extrapolation of the conclusion. In addition, our study was not a complete cohort study, and the causal order relationship identified was not the strongest. We focused only on the relevant indicators of CVDs rather than the disease itself. Future research is needed to further confirm the causal relationship between MDTs and CVDs. The major strengths of the study are that we used trajectories to show the long-term MD pattern and included various CVD-related risk factors in one study.

## 5 Conclusion

Four MD trajectories were found among the total participants, both men and women, which were summarized as persistently low MDT, ascent MDT, descent MDT, and persistently high MDT. Adherence to a high MDT may reduce the level of certain CVD-related risk factors. The effect of the remote MD on CVD-related risk factors may be greater than the effect of the proximal MD.

## Data Availability

The original contributions presented in the study are included in the article/Supplementary material, further inquiries can be directed to the corresponding authors.
