# Supplementary material for: Relationships between different trends of the Mediterranean diet and cardiovascular disease-related risk factors in China: results from the CHNS study, 1997–2009
Source: Front Nutr. 2024 Nov 8;11:1463947. doi: 10.3389/fnut.2024.1463947 (PMC11583441; doi:10.3389/fnut.2024.1463947)
Supplement: Supplementary file 1 [file Table_1.DOCX]

**Table S1** Estimation process for the trajectory groups of Mediterranean diet

| Number of Groups | Polynomial | BIC | | |
| --- | --- | --- | --- | --- |
|  |  | All | Males | Females |
| 1 | 3 | -32819.31 | -15422.98 | -17393.13 |
| 2 | 3 3 | -32619.68 | -15324.71 | -17326.41 |
| 3 | 3 3 3 | -32589.07 | -15321.06 | -17320.11 |
| 4 | 3 3 3 3 | -32595.52 | -15335.53 | -17332.95 |
| 5 | 3 3 3 3 3 | -32616.48 | -15356.66 | -17353.78 |
| 4 | 1 1 2 3 | -32324.72 |  |  |
| 4 | 1 1 1 1 |  | -15198.40 |  |
| 4 | 1 1 1 3 |  |  | -17160.77 |
| Abbreviation: BIC, Bayesian information criterion. | | | | |
