# Supplementary material for: Relationships between different trends of the Mediterranean diet and cardiovascular disease-related risk factors in China: results from the CHNS study, 1997–2009
Source: Front Nutr. 2024 Nov 8;11:1463947. doi: 10.3389/fnut.2024.1463947 (PMC11583441; doi:10.3389/fnut.2024.1463947)
Supplement: Supplementary file 2 [file Table_2.DOCX]

**Table S2** Model evaluation indexes of three Mediterranean diet trajectories

| Gender | Trajectory | APPA | OCC | EGP (%) | AGP (%) |
| --- | --- | --- | --- | --- | --- |
| All | Group 1 | 0.66 | 23.47 | 7.59 | 6.74 |
|  | Group 2 | 0.55 | 6.02 | 16.74 | 12.40 |
|  | Group 3 | 0.56 | 8.38 | 13.37 | 10.48 |
|  | Group 4 | 0.82 | 2.67 | 62.31 | 70.38 |
| Male | Group 1 | 0.67 | 27.21 | 6.91 | 6.07 |
|  | Group 2 | 0.55 | 6.70 | 15.38 | 11.69 |
|  | Group 3 | 0.58 | 6.87 | 16.55 | 13.26 |
|  | Group 4 | 0.81 | 2.74 | 61.17 | 68.98 |
| Female | Group 1 | 0.65 | 23.68 | 7.32 | 6.21 |
|  | Group 2 | 0.54 | 6.24 | 15.94 | 12.15 |
|  | Group 3 | 0.57 | 8.52 | 13.25 | 9.62 |
|  | Group 4 | 0.82 | 2.59 | 63.50 | 72.03 |
| Abbreviation: APPA, Average posterior probability of assignments; OCC, odds of correct classification; EGP, estimated group proportions; APG, assigned group proportions. | | | | | |
